# Comprehensive assessment of intelligent unmanned vehicle techniques in pesticide application: A case study in pear orchard

**DOI:** 10.3389/fpls.2022.959429

**Published:** 2022-08-23

**Authors:** Yulin Jiang, Xiongkui He, Jianli Song, Yajia Liu, Changling Wang, Tian Li, Peng Qi, Congwei Yu, Fu Chen

**Affiliations:** ^1^College of Science, China Agricultural University, Beijing, China; ^2^College of Agricultural Unmanned System, China Agricultural University, Beijing, China; ^3^Key Laboratory of Farming System, Ministry of Agriculture and Rural Affairs, College of Agronomy and Biotechnology, China Agricultural University, Beijing, China

**Keywords:** plant protection, UAV, UGV, application performance, ecological assessment, orchard

## Abstract

The intelligent pesticide application techniques in orchards have grown rapidly worldwide due to the decrease in agricultural populations and the increase in labor costs. However, whether and how intelligent pesticide application techniques are better than conventional pesticide application remains unclear. Here, we evaluated the performance of the unmanned aircraft vehicle (UAV) and unmanned ground vehicle (UGV) on pesticide application, ecological environment protection, and human’s health protection compared to conventional manual methods. We quantified characteristics from the aspects of working effectiveness, efficiency, environmental pollution, water saving and carbon dioxide reduction. The results showed that the UAV application has the advantages of a higher working efficiency and less environmental pollution and natural resource consumption compared to the UGV and conventional manual methods despite of its worse spray performance The UGV application techniques could improve spray performance at the cost of high environmental pollution. The conventional spray gun technique was unfriendly to environmental and resource protection although it showed a better spray performance. Thus, the balance of improving spray performance and controlling environmental pollution is the key to improve the performance of UAV and UGV technology in the future. The study could be useful in the development of intelligent pesticide application techniques and provide scientific support for the transition of intelligent management in orchards.

## Introduction

Orchard area and fruit production have rapidly increased to meet the higher demand for fruit consumption over the past decades. The global orchard area and fruit production increased by approximately 22 and 54%, respectively (FAO, 2020). As the country with largest population in the world, China has the largest orchard area and the highest fruit production in the world (Jiang et al., 2021). The fruit industry is not only an advantageous industry in China but also a labor-intensive industry. Plant protection is an important part of orchard management with high labor demands (He et al., 2017). However, rapid urbanization during past decades has led to severe labor shortages in the orchard industry, and the challenges associated with the aging population in the county is becoming increasingly prominent (Zhao et al., 2021). At present, most management of orchard pesticide application relies on manual operation, which is characterized by high labor intensity, low efficiency and low standardization (Zhai et al., 2018; Liu et al., 2021). Moreover, pesticide application has potential damage both in human and environment (Pan et al., 2020; Cai et al., 2021). In general, green and sustainable development is an important objective of global agricultural transformation (Abbas and Sagsan, 2019; Guo et al., 2020). Cleaner production with a reduction in greenhouse gas emissions and resource consumption in agricultural management is urgent and important based on global climate change (Young et al., 2015). How to strengthen ecological environmental protection during the utilization of pesticides in orchard is a key issue in national development plans (Li et al., 2022). In addition, legislation and ethics have to go hand in hand when considering the design of legal solutions due to the value-laden nature of the concerns associated with robotic systems (EU, 2016; Benos et al., 2022).

Generally, the air-assisted spray method (a machine with pump and air-assisted equipment) and the human spray gun method (a machine with pump and several spray guns) are widely used in orchard plant protection (Khot et al., 2012; Lu et al., 2021). The former method has been recognized as a high-efficiency pesticide application technology and is widely used for pest control in orchards (Li et al., 2022). However, it is only suitable in standardized orchards with fixed wide spacing and relatively flat pavement (Wang et al., 2022). Human spray guns can make up for this shortcoming. However, high labor costs limit its application in large-scale orchard management (An et al., 2020). To solve these problems, intelligent pesticide application technology can be an alternative choice in orchard plant protection, which has been growing rapidly worldwide as a new method for the application of plant protection products, especially in East Asian and Southeast Asian countries (He et al., 2017; He, 2018). Unmanned aircraft vehicles (UAVs) and unmanned ground vehicles (UGVs) are two major kinds of intelligent equipment that have been widely adapted for agricultural management (Kefauver et al., 2017; Zhang et al., 2019). Intelligent pesticide application technology fits the current development requirements of modern agriculture: high efficiency, high quality and economically efficient as well as standardization and informatization (Wang et al., 2016; Lan and Chen, 2018). It has a major advantage in low labor demand, which is important for orchard management in the future. Moreover, intelligent equipment can also ignore terrain obstacles and planting patterns, which is important for orchards in hilly areas and disorderly planting orchards, such as orchards in southwestern China (Wang et al., 2022). Some intelligent equipment has the ability to work at night, which can significantly improve working efficiency. Finally, the outstanding progress in vision sensors in conjunction with that of machine learning has allowed the sustainable targeted application (Benos et al., 2021).

A comprehensive evaluation of the stability and effectiveness of intelligent pesticide application techniques during actual operation scenario is important for technological improvement and popularization; however, this exploration is still limited. Many studies have focused on parameter optimization in specific equipment. For example, the influence of operating techniques on the spray effect through ground machine application, including travel speed, nozzle type, and spray pressure, has been explored by many researchers (Nuyttens et al., 2007; Li et al., 2021a, 2022; Grella et al., 2022). Similarly, flight height and velocity, tree shape, UAV type and drift have been widely studied for UAV application (Tang et al., 2018; Meng et al., 2020; Wang et al., 2021). On the other hand, previous studies have also focused on equipment design and remolding to improve application performance (Li et al., 2017; He, 2019). These studies were meaningful for improving the equipment and application effectiveness. However, it is difficult to achieve farmer recognition. Previous study showed that farmers from larger farms focus more on financial benefits from robots and prefer large autonomous tractors. Conversely, small-scale or organic farmers consider environmental benefits of field crop robots relatively more important and favor small robots (Spykman et al., 2021). Few studies have focused on the effects of different pesticide application techniques on the ecological environment. It was essential to carry out a comprehensive comparison between conventional and intelligent technology and evaluate the difference between different pesticide application techniques in terms of both economic and ecological benefits. Some studies have compared UAV application methods to air-assisted spray methods in terms of spraying performance (Sarri et al., 2019; Martinez-Guanter et al., 2020; Li et al., 2021a). However, most studies were concentrated in a small area (less than 0.1 ha), which is quite distinct from the actual operation situation. On the other hand, the ecological characteristics of separate application techniques, such as environmental pollution, resource consumption and greenhouse gas emission, should also be evaluated due to the national green and sustainable development strategy in agricultural management, but it is still limited.

Thus, to understand the comprehensive performance of intelligent pesticide application technology and conventional technology in orchards, a comparison study through field positioning experiments was conducted. This study aimed to clarify the characteristics of different pesticide application techniques from the aspects of working effectiveness, efficiency, environmental pollution, water savings and CO_2_ reduction and to put forward suggestions for the development of orchard plant protection in the future, providing scientific support for the transition of intelligent management in orchards.

## Materials and methods

### Study area

**Figure 1:**
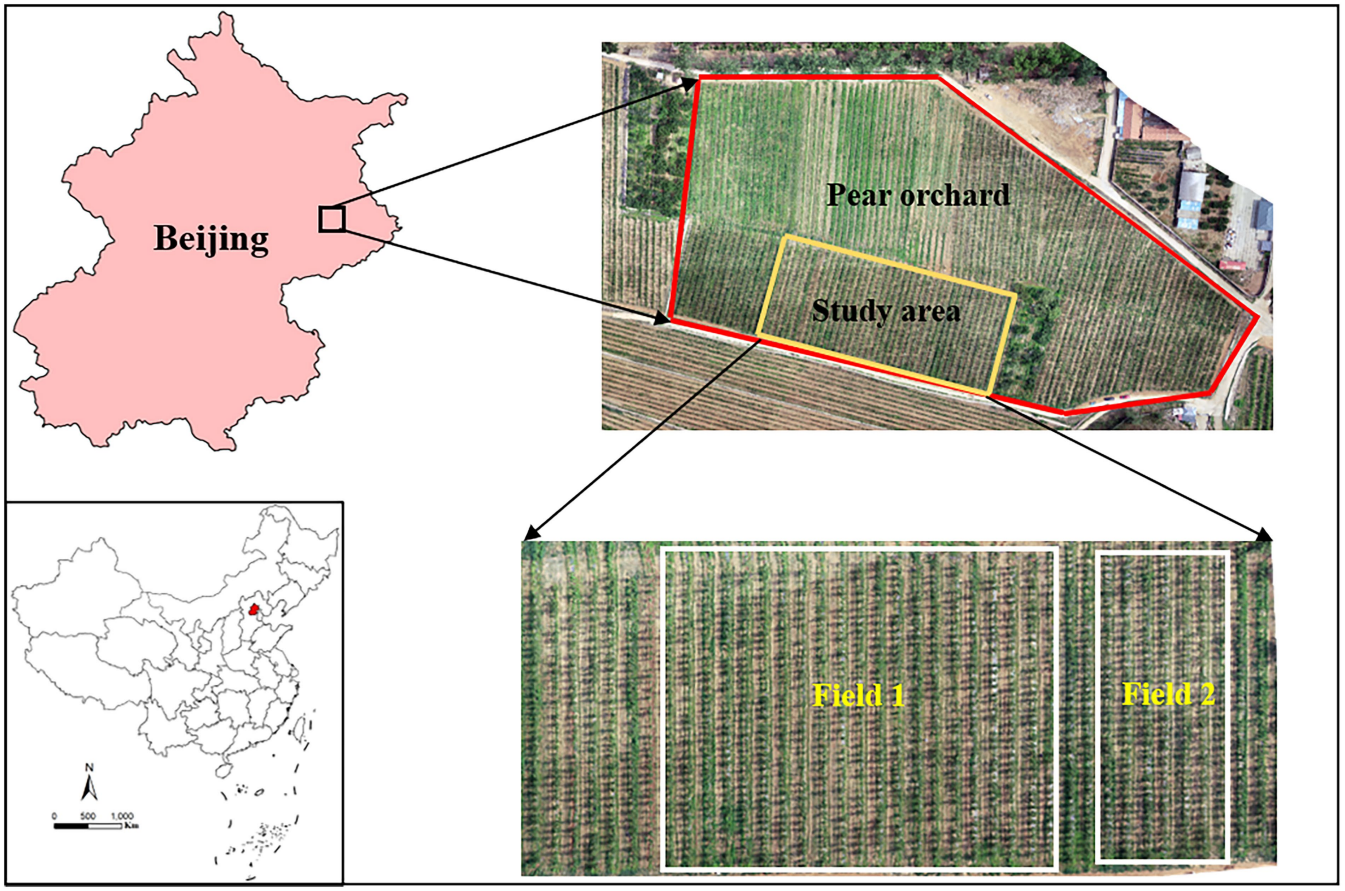
Location of the study area and test fields. Field 1 represents the test field of the unmanned aerial vehicle and ground machine spraying method; Field 2 represents the test field of the conventional artificial method.

A pear (*Pyrus bretschneideri*) orchard was selected as the target study area, which was located in Pinggu district, Beijing (Figure 1). The 4-year-old pear orchard covered an area of approximately 13 hectares. The dwarfing and dense planting mode was adapted in the target pear orchard. The distances between rows and trees were 4 and 1.5 m, respectively. The average height of the trees was approximately 3 m. Tests were conducted during October 2021. The daily daytime temperature ranged from 18 to 25°C, while the humidity was 45%. The wind speed was below 1 m/s during the experiment.

### Sprayer characteristics

**Figure 2:**
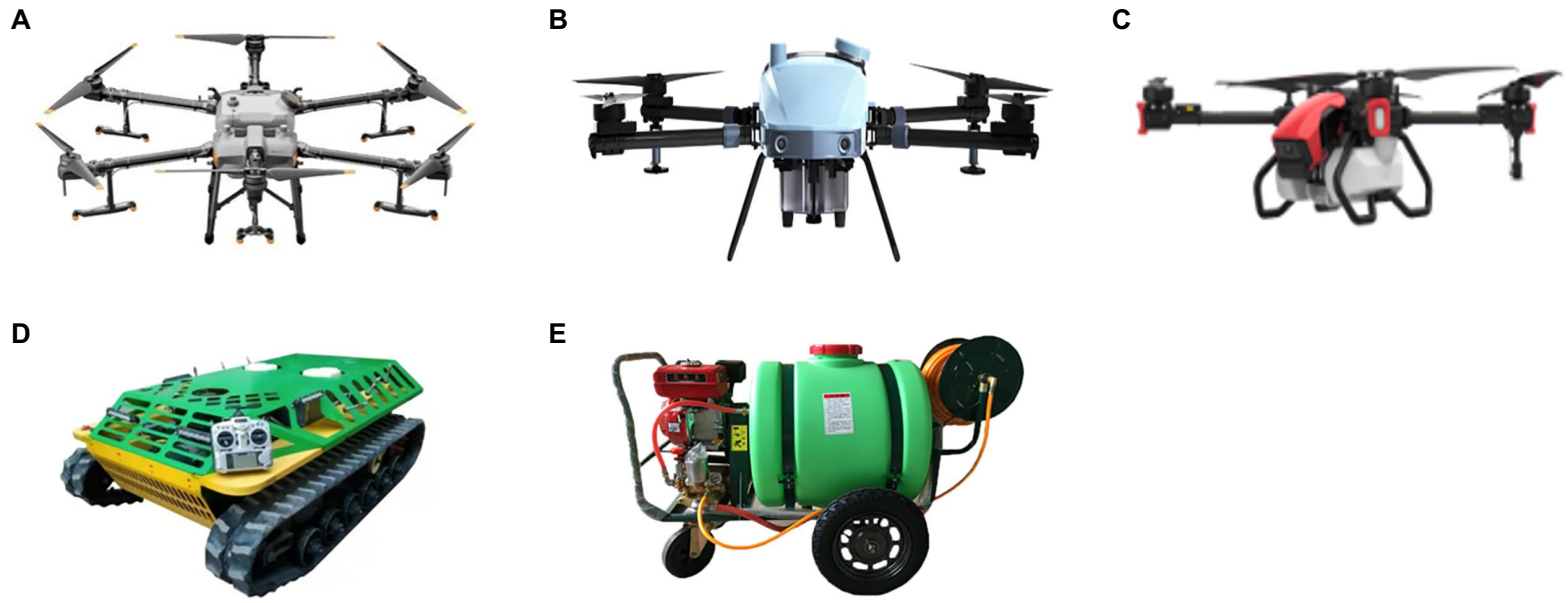
Major pesticide application equipment used in the study, including unmanned aircraft vehicles **(A–C)**, unmanned ground vehicles **(D)** and conventional manual spray guns **(E)**.

Five orchard pesticide application equipment were adapted in the study, including three mainstream orchard unmanned aircraft vehicles, one unmanned ground vehicle and one conventional manual spray gun (Figure 2). The basic parameters of the different equipment are shown in Table 1. All treatments were modeled on the actual operation parameter environment, which was promoted by the guidance of local orchardists and professional operators.

**Table 1:** Equipment and application parameters of unmanned aircraft vehicles (UAV), unmanned ground vehicle (UGV) and conventional manual spray guns (CONV) used in the trials.

Parameter	UAV-1	UAV-2	UAV-3	UGV	CONV
Tank capacity	30 l	20 l	40 l	200 l	300 l
Working speed	1.1 m/s	1.5 m/s	2.6 m/s	1 m/s	3.5 m/min
Spray width	7 m	3.5 m	3.2 m	6–8 m	10–12 m
Flight height	4.5 m	3.3 m	5.5 m	–	–
Flow rate	2.98 l/min	3.01 l/min	2.82 l/min	8 l/min	22 l/min
Engine power	7.2KW	7.2KW	7.2KW	9.5KW	4.8 KW

The engine power of the UAV represents the battery charge engine power.

### Experimental design

Three treatments were set in this study: spraying by unmanned aircraft vehicle (UAV), spraying by unmanned ground vehicle (UGV) and spraying by conventional spray gun (CONV). The average level of three major UAVs was adapted in order to represent the common performance about UAV application techniques. To better simulate the actual working environment, a 1 ha test field was set in the T1 and T2 treatments, which contained approximately 40 rows of pear trees. The large working area included at least 1 battery change or water refill. Considering the relatively low working efficiency in conventional manual application, a 0.08 ha test field was set in T3, which contained 5 rows of pear trees. Three types of unmanned aircraft vehicles were adapted for the test, and two repetitions were set for each piece of equipment. Meanwhile, both the T2 and T3 treatments were repeated 3 times. All intelligent application equipment were operated manually by professional operator and tried to keep uniformity in repetition. Through positioning experiments, the characteristics of different pesticide application techniques in spraying performance, working efficiency, environmental pollution and resource consumption were comprehensively analyzed.

#### Spray effectiveness test

**Figure 3:**
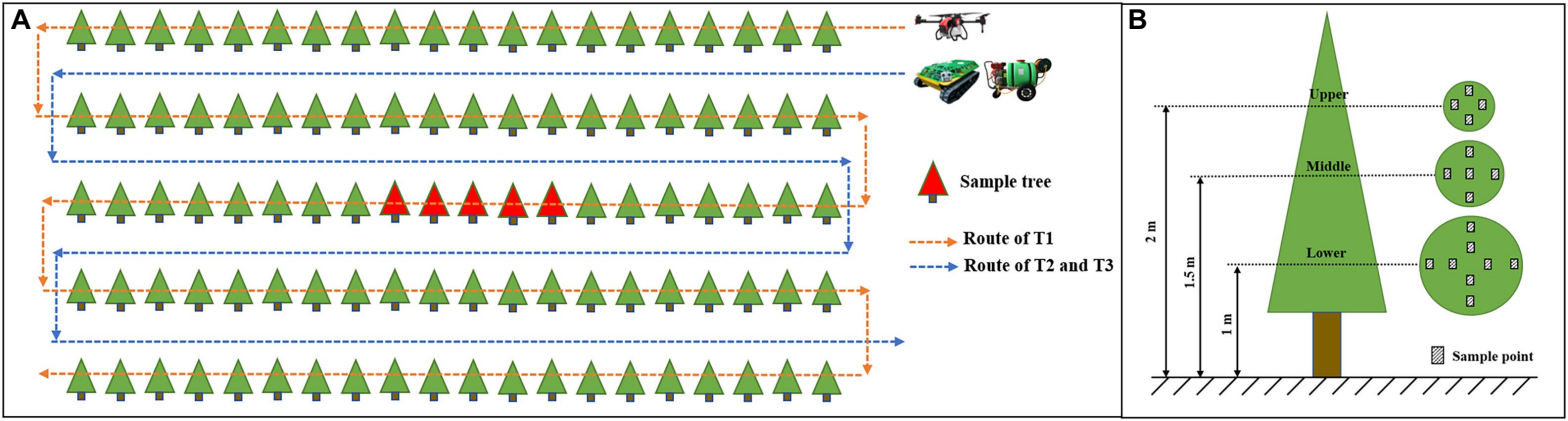
Layout of the test **(A)** and sample point **(B)**. The yellow line represents the flight route of the UAV; the blue line represents the route of UGVs and humans; the red triangle represents the sample trees; and the black rectangle represents the sample point.

Five consecutive trees were sampled in the middle row of each test field, and sample trees were no less than 10 m from the beginning and end of the row to ensure that the equipment was working stably when passing through (Figure 3A). The sample tree was divided into three layers, including upper, middle and lower layers, and the height of each layer was 2, 1.5, and 1 m, respectively. The number of sample points set in the upper, middle and lower layers was 4, 5 and 8, respectively (Figure 3B). The well-grown leaf which was fully expanded was chosen as the sample leaf, and 2 white art papers (60 mm × 40 mm) were attached on both the adaxial side and abaxial side of it. Ponceau 4R, a kind of food coloring with no risk of environmental pollution and human damage, was added as a replacement for pesticide in the test. All samples were scanned with a scanner (DS-1610, Epson, Beijing, China) at 400 dpi to obtain images. ImageJ, an image processing program, was used for the analysis to obtain spray coverage (Zhu et al., 2011).

The deposit coverage on both the adaxial side (CAD) and abaxial side (CAB) of the leaf was calculated in different layers during the test. The ratio of deposit coverage on the abaxial side and whole leaf (RBW) was calculated to account for droplet distribution uniformity on leaves (Li et al., 2022). The coefficient of variation, CV (%) was also calculated in the study.


(1) $$RBW=\frac{CAB}{CAB+CAD}$$

(2) $$CV=\frac{SD}{\bar{X}}\times 100\%$$

where SD represents the standard deviation of each treatment and *x* represents the mean value of each treatment.

#### Environmental pollution test

**Figure 4:**
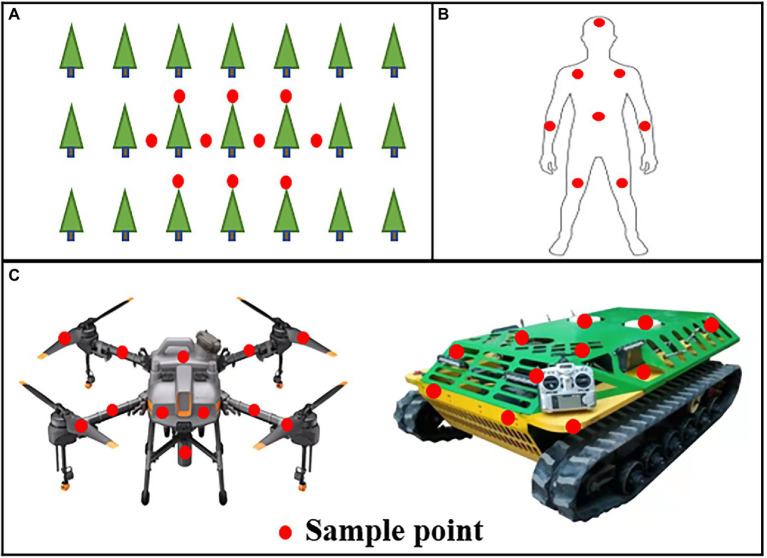
Layout of the sample site in the test of ground residue **(A)**, human body residue **(B)** and machine residue **(C)**. The red circle represents the sample point.

Pesticide application pollution mainly includes ground residue, machine residue and human body residue (Musiu et al., 2019; Rani et al., 2021). Deposit coverage was used to evaluate the degree of pollution in different positions. For the ground residue test, white art papers were attached to the ground at the center of rows and trees (Figure 4A). For the machine residue test, white art papers were randomly attached around the equipment, including the rotor, arm, tank, and shell (Figure 4C). For the human body residue test, white art papers were randomly pasted on the head, arm, chest and leg (Figure 4B). All samples were also scanned with a scanner (DS-1610, Epson, Beijing, China) at 400 dpi to obtain images, and ImageJ was used for the analysis to obtain average spray coverage.

#### Working efficiency test

Working efficiency (WE, ha/h) referred to the area of application completed in unit time. This study provides significant guidance for orchard management between different application techniques. The calculation formula was as follows:


(3) $$\text{WE}=\frac{A}{\sum_{i=1}^{n}T_{i}}$$

where *T_i_* represents the overall application time in process *i* (h). The process includes dosing, battery change and machine transfer. *A* represents the application area (ha). In this study, stopwatch was used to record the progress of application. For UAV application, time recording was initiated at the route planning and ended at the complement of the last line of trees. For the ground machine and conventional manual techniques, time was started at dosing and ended at the complement of the last line of trees. Considering the difference in the application area between treatments, the application area per unit time was uniformly converted.

#### Water consumption test

Orchard workers usually use a fixed amount of pesticide, although the application technique varies. Thus, we calculated the overall water consumption among the different treatments. The calculation formula was as follows:


(4) $$W=\sum_{i=1}^{n}W_{b}-W_{e}$$

where *W* represents the overall water consumption in the test (L). *W_b_* represents the volume of water in the beginning (L); *W_e_* represents the volume in the end (L); and *n* represents the time of dosing. The average water consumption per unit area was uniformly converted.

#### Machine CO_2_ emission test

Machine CO_2_ emissions (E_CO2_, kg) refer to the CO_2_ directly generated by the combustion of gasoline used in agricultural production. The emission was equal to the amount of gasoline multiplied by the CO_2_ emission coefficient of gasoline. The calculation formula was as follows:


(5) $$E_{C02}=\frac{\left(G_{b}-G_{e}\right)}{A}\times 2.9251$$

where *G_b_* represents the gasoline in the beginning and *G_e_* represents the gasoline in the end, kg; *A* represents the application area, ha. The CO_2_ emission coefficient of gasoline in China was 2.9251, kgCO_2_-eq/kg. The determination of the CO_2_ emission coefficient was referred from Intergovernmental Panel on Climate Change (IPCC) guidelines for greenhouse gas inventory and provincial guidance for greenhouse gas inventory complications (Paustian et al., 2006; Yan et al., 2022). In the UAV application treatment, the oil consumption of the generator used for battery charging was mainly recorded. Gasoline consumption of the engine was recorded in the UGV treatment while gasoline consumption of the pump was recorded in the conventional spray gun treatment.

### Data processing and analysis

Feature normalization was adapted in the study to make different types of data in the same range. StandardScaler was one of the feature normalization methods which had been widely adapted in data processing. After normalization, the mean value of each column of the matrix was 0 and the standard deviation was 1. The calculation formula was as follows:


(6) $$X=\frac{\left(x-\mu \right)}{\sigma }$$

where *X* represents the result after feature normalization; *x* represents the initial data; *μ* represents the average value of dataset; *σ* represents the standard deviation of dataset.

All statistical analysis were conducted by SPSS 26.0 software (IBM Crop., Armonk, NY, United States). Before the statistical tests, assumptions of normality and homoscedasticity of the datasets were tested using the Shapiro–Wilk and Levene tests, respectively. One-way analysis of variance was applied to test the effects of different pesticide application techniques on spray performance, environmental residue, working efficiency and resources consumption. Significant differences between various treatments were identified by the least significant difference test at the *p* < 0.05 level. Data visualization was performed using the R package ggplot 2.

## Results and discussion

### Assessment of spraying performance

Various deposit coverages occurred in different layers between different spraying techniques (Table 2). The average deposit coverages of UAV, UGV, and CONV in the test were 3.4, 60.3 and 34.9%, respectively. Similar to previous studies, deposit coverage on the adaxial side was higher than that on the abaxial side in all treatments (Grella et al., 2020; Salcedo et al., 2020). According to the UAV’s low-volume-spray characteristics, the deposit coverage in UAV was significantly lower than that in the other treatments. The average deposit coverage was approximately 3.4%. The deposit coverage decreased from the upper to lower layer on both the adaxial and abaxial side. The deposit coverage on the abaxial side of the lower layer was approximately 1.1%. In addition, the average CV of UAV (66.2%) was higher than that of the other treatments. This result indicated that UAV application technology is unstable, which could affect the overall pest control in the orchards. Generally, it could achieve pest control effects when the deposit coverage exceeds 1% during UAV application (Wang et al., 2022) due to its high-concentration spraying property. The results indicated that UAV application could adapt to orchard pest control. However, unstable effectiveness would reduce acceptance for farmers because of the uncertainty in the control effect. Previous research also showed mediocre performance in UAV spraying (Li et al., 2021c). In contrast to field crops, pest control in orchards is stricter because it will affect the economic benefit significantly.

**Table 2:** Comparison of deposit coverage on the adaxial side (CAD) and abaxial side (CAB) of the leaf between different layers and spraying techniques.

Layer	Treatment	CAD		CAB	
		Mean (%) ± SE	CV (%)	Mean (%) ± SE	CV (%)
Upper	UAV	6.8 ± 1.6c	66.4	1.6 ± 0.2c	46.2
	UGV	51.8 ± 6.4a	41.1	34.3 ± 8.9a	48.3
	CONV	23.1 ± 4.2b	42.3	16.2 ± 6.5b	46.5
Middle	UAV	5.7 ± 0.9c	63.5	1.4 ± 0.9c	65.7
	UGV	79.6 ± 5.6a	27.0	42.2 ± 6.9a	59.2
	CONV	47.4 ± 3.7b	19.6	33.3 ± 8.3ab	45.5
Lower	UAV	3.2 ± 0.2c	28.5	1.1 ± 0.2c	57.5
	UGV	91.1 ± 3.1a	17.5	62.6 ± 4.8a	40.3
	CONV	52.6 ± 7.8b	17.9	36.7 ± 5.4b	42.6

Mean represents average values of 18–27 biological replicates. ± indicates standard error of each dataset. The different letters in the same column indicate significant differences at the *p* < 0.05 by ANOVA test with LSD as *post hoc* test.

Unmanned ground vehicle application technology showed the highest deposit coverage on both the adaxial and abaxial sides of leaves among all treatments. In contrast to the UAV, the deposit coverage increased from the upper to the lower layer. This was mainly because the ground machine sprays from the bottom to the top through high-pressure assistance. It had a relatively low average CV (37.1%) in different layers, which is more stable than UAV application. Generally, deposit coverage of approximately 30–70% in ground pesticide application equipment is normal, and some high-application-volume operations even approach 100% (Gil et al., 2021; Wang et al., 2022). Although ground machines have better performance in pesticide application, high environmental pollution and pesticide residues should be given more attention.

Conventional spray gun technology showed similar characteristics to UGV in different layers. The deposit coverage in each layer was lower than that in ground machine technology, and the average CV was 42.2%. The results showed that the conventional method has satisfactory performance in orchard plant protection. However, human application is uncertain. It depends on the experience of the farmers, degree of fatigue and other factors associated with the farmers, which could directly affect the performance of plant protection in practice (Foque et al., 2012). At present, there is no deposit coverage standard for fruit trees, and it is difficult to compare the performance of different application techniques. The perspectives of uniformity and penetration are usually selected as indices of application evaluation (Wang et al., 2022). For further study, a reasonable assessment index system should be built through big data surveys and multipoint experiments.

### Assessment of environmental pollution

**Figure 5:**
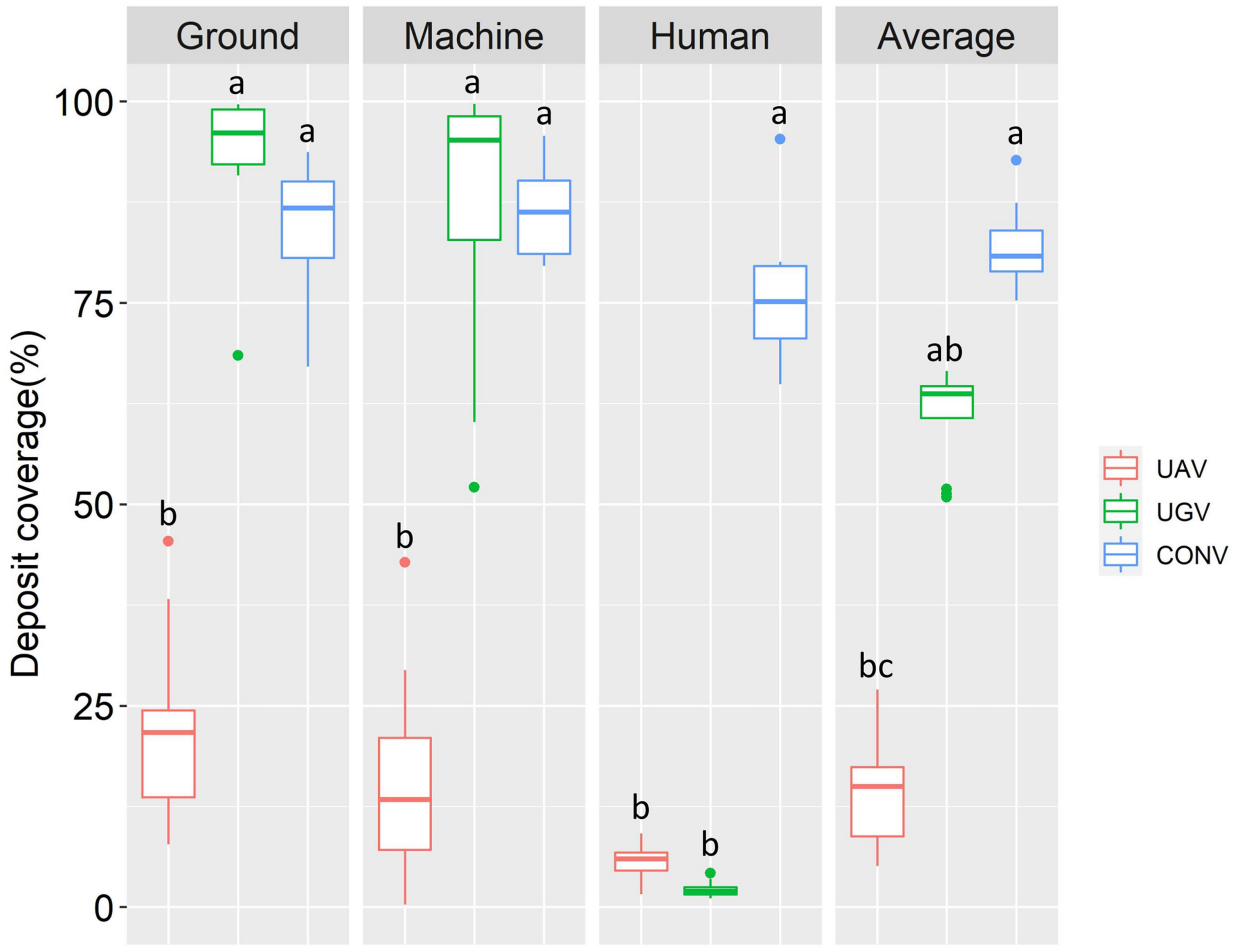
The droplet residue on the ground, machine and human body of unmanned aircraft vehicle technique (UAV), unmanned ground vehicle technique (UGV) and conventional manned technique (CONV). Different letters in the same column indicate significant differences at the *p* < 0.05 level under ANOVA test with LSD as *post hoc* test. UAV had 18 biological replicates while UGV and CONV had 13 biological replicates.

Deposit coverage on the ground, machine and human body were estimated in the study, which could represent the environmental pollution in different spraying technologies. The average level of environmental pollution during pesticide application was CONV > UGV > UAV (Figure 5). UAV showed a relatively lower environmental residual than the other treatments. Deposit coverage on the ground, machine and human body were 22, 15 and 6%, respectively. Although UAV application technology uses a low-volume spraying method, it can still cause ground residue in actual applications. Drift of droplets could lead to uncertainty in pesticide application (Wang et al., 2021). On the other hand, a suitable flight route is also important to in the application of a sprayer above the canopy of the tree. Machine pollution of UAV application technology mainly came from the interaction of air flow and environmental wind, which allow droplets to drift to the surface of the machine. Human body pollution in UAV application could also come from droplet drift. Drift characteristics increase the uncertainty of environmental pollution in agricultural UAV applications (Liu et al., 2020; Martinez-Guanter et al., 2020). It would be harmful to the surrounding environment when we use UAV technology in agricultural management, such as pesticide and herbicide application.

High ground residual and machine residual values occurred in UGV, which were significantly higher than those in UAV. The deposit coverages of UGV on the ground and machined were 93 and 88%, respectively. High-volume spraying under UGV technology could achieve better performance on leaves at the cost of environmental pollution. It also demonstrated that the pesticide utilization efficiency of UGV application technology could be improved, which was meaningful for green and sustainable development in agricultural management. However, UGV showed a lower residual on the human body, with an of average 2% coverage. The remote operation using in UGV technology could effectively reduce the exposure of humans in the application environment and protect operators from pesticide damage.

Conventional spray gun showed the highest environmental pollution compared to UAV and UGV. The deposit coverage on the ground and machine were close to UGV. Human body residue amount was significantly higher than others, which could lead to greater potential threat to the operator. It is easy to understand that operators were fully exposed to the application environment, which could result in a large amount of residue on the body. Operator health has received increasing social attention because of the high operator exposure, which has been accused of negative health issues such as respiratory, dermatological, neurological, reproductive, endocrine, and gastrointestinal diseases (Nuyttens et al., 2009). Conventional pesticide application technology was not sustainable unless personnel safety was addressed.

Generally, the results indicated that intelligent plant protection technology could reduce environmental pollution and human damage compared to conventional methods. However, there is still much room for improvement to reduce the ground and machine residue to further increase the pesticide utilization efficiency. For UAV application technology, drift influence should be evaluated both in theory and in practice. Meanwhile, for UGV application technology, volume control and spray angle improvement may be useful for the reduction in environmental pollution.

### Assessment of working efficiency

Working efficiency could directly affect cost estimation and technology promotion. The results showed that the working efficiency of UAV application was approximately 1.82 ha/h, which was the highest among all treatments (Table 3). This result was lower than data recommended from UAV companies or some previous studies. However, these studies did not consider the time of dosing, charging, or changing the battery. The properties are very important in practice. The duration of a single flight of a UAV was usually no more than 20 min, and it needed to be maintained through battery replacement in large-scale operation. Although each battery replacement and charging time could be shortened by engaging experienced workers, multiple uses during application would result in longer time of application. Meanwhile, all UAV operations should be carried out on the ridge, and the round-trip operation also requires time. The results in this study could be closer to the actual application.

**Table 3:** Comparison of the working efficiency and resource consumption of different spraying technologies.

Treatment	Area (ha)	Time (h)	Efficiency	Water usage	Gasoline usage	CO_2_ emission
			ha/h ± SE	L/ha ± SE	L/h ± SE	kg/ha ± SE
UAV	1.00	0.55	1.82 ± 1.1a	105 ± 1.1c	3.00 ± 0.5a	3.60 ± 0.5b
UGV	1.00	0.78	1.28 ± 0.9b	360 ± 6.2b	1.52 ± 0.4b	3.15 ± 1.3b
Conventional	0.08	0.84	0.09 ± 0.01c	3,375 ± 101.9a	1.48 ± 0.2b	39.00 ± 4.2a

All values in table represent average values of 3–9 biological replicates. ± indicates standard error of each dataset. Different letters in the same column indicate significant differences at the *p* < 0.05 level under ANOVA test with LSD as *post hoc* test.

The working efficiency of UGV application following UAV was approximately 1.29 ha/h. The results might be higher because the test field was in a standardized orchard, which was easier for UGV working. The flat terrain allowed the ground machine to move at a constant speed, and rational planting allowed the machine to turn around more easily. However, in some disorderly planting orchards, the ground machine often needs to return to the original path and then proceed to the next row. For some hilly orchards, the moving speed could also be difficult to control, which would affect the working efficiency of the intelligent ground machine.

Conventional technology showed the lowest working efficiency compared to others, which was approximately 0.09 ha/h. The result could be lower than actual application because of the efficiency of a single operator. In most orchard pesticide applications, there are usually at least two people working simultaneously using spray gun technology. One pump could connect to 4 spray guns at the same time. Thus, the actual working efficiency was difficult to calculate considering different orchard scales or management. However, compared with intelligent application technology, working efficiency assessment based on a single-person working environment is still meaningful.

In general, the higher working efficiency that occurred in the UAV and UGV applications showed the advancement of intelligent pesticide application techniques. The working efficiency of the UAV was almost 20 times higher than that of the conventional method. UGV could also improve efficiency, which was approximately 14 times that of conventional technology. Due to the shortage of the agricultural population and the increase in labor costs, intelligent pesticide application technology will be an alternative in the future due to its significantly high efficiency.

### Assessment of water-saving potential

The economical utilization of resources is also an important aspect for the comparison of different pesticide application techniques. The results showed that the variation characteristics of water consumption among the different treatments were UAV < UGV < Conventional (Table 3). The average water usage in UAV application was approximately 105 l/ha. The lower water consumption was mainly due to its low-volume and high-concentration spraying characteristics. The water consumption under UGV application was approximately 360 l/ha, which was almost three times higher than that under UAV application. The water consumption of conventional technology was approximately 3,375 l/ha, which was significantly higher than that of the other treatments. The results indicated that intelligent plant protection equipment has a significant advantage in water savings during pesticide application compared to conventional methods, especially in UAV application technology. New spraying technology could save approximately 3,000 l/ha water resources within a single-time application. Generally, orchards usually need at least 8 pesticide applications during the growing season. The adaptation of intelligent application technology could have vast water saving potential in orchard management. Meanwhile, in actual orchard plant protection, farmers usually adapt the same amount of pesticide regardless of the application technique to ensure control effectiveness. Thus, current intelligent application techniques cannot reduce pesticide consumption. However, with the development of variable pesticide application technology, it will be possible to achieve both water and pesticide savings through intelligent techniques in the future (Chen et al., 2021).

### Assessment of CO_2_ emission reduction potential

UAV application showed higher gasoline consumption compared to UGV and conventional technology. The average gasoline consumption of UAV application was approximately 3 l/h, while gasoline consumption of UGVs and conventional technology was 1.52 and 1.48 l/ha, respectively (Table 3). UAV technology was recognized as cleaner energy equipment because of replacing fuel with electric power (Matlock et al., 2019). However, in practical operation, UAV application needs to maintain its endurance through long-term battery charging, which still requires a high amounts of gasoline consumption of gasoline. The results showed that the gasoline consumption of UAV application in fixed time was twice that in UGV application and the conventional method. The average CO_2_ emissions of machines through the combination of working efficiency and gasoline usage were also calculated in the study. The average CO_2_ emissions of the UAV, UGV and conventional techniques were 3.60, 3.15 and 39.00 kg/ha, respectively. Although UAV application consumes more gasoline in a fixed time, high working efficiency could eliminate its negative effect on CO_2_ emissions to a certain degree. In contrast, conventional application technology has much higher CO_2_ emissions due to its low working efficiency. UGV application showed better performance in reducing CO_2_ emissions than the other treatments. The results indicated that intelligent pesticide application technology could effectively reduce CO_2_ emissions from the machine itself compared with the conventional method. The application of intelligent techniques in orchards is of great significance to the national carbon neutrality strategy.

## Perspectives and implications

**Figure 6:**
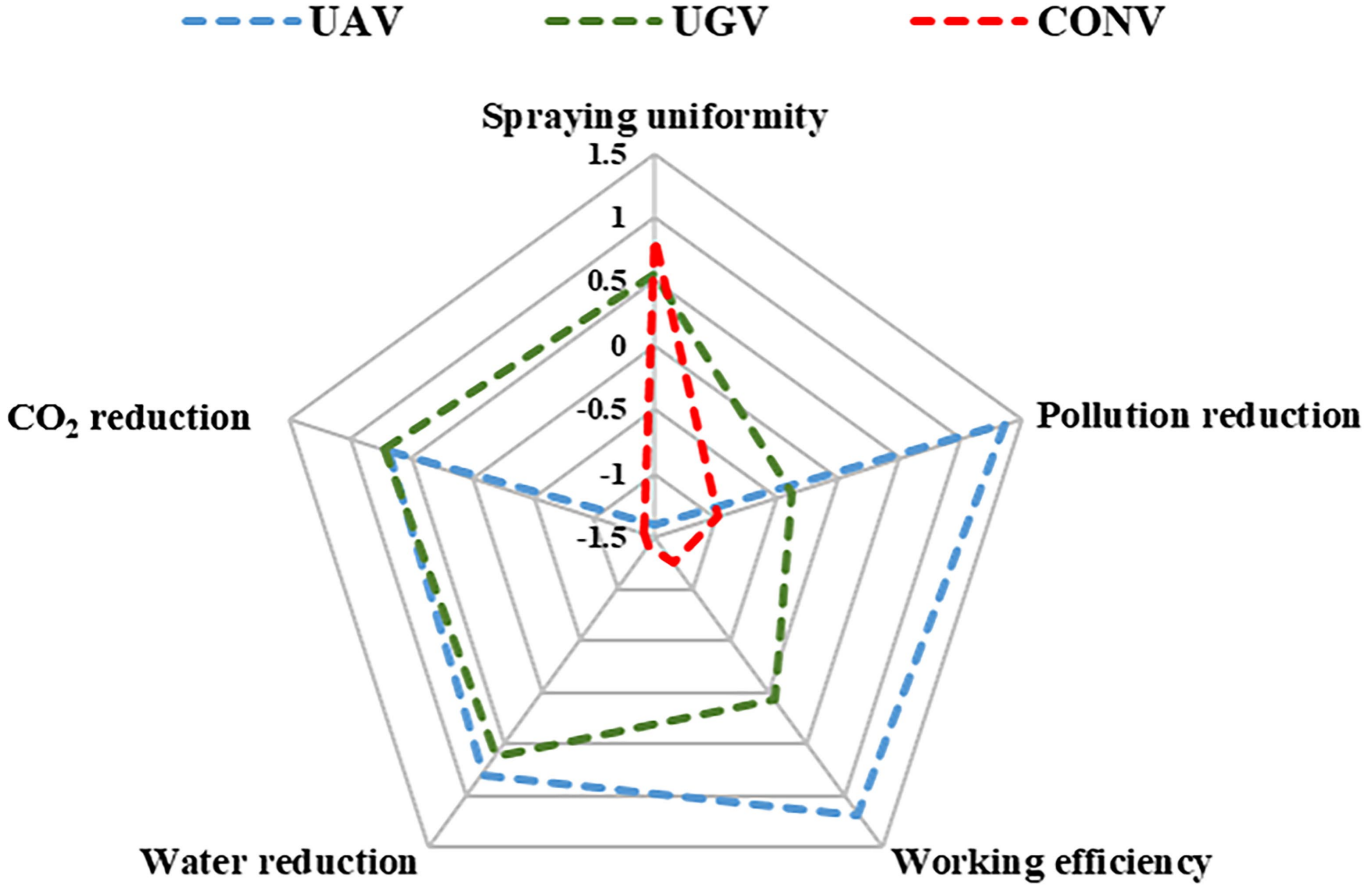
Comprehensive comparison of characteristics in different pesticide application techniques. Blue line represents unmanned aircraft vehicle technique (UAV), green represents unmanned ground vehicle technique (UGV) and red line represents conventional manned technique (CONV). Data was normalized and 0 represents the average value of each column. Positive values represent performance above average in each column whereas negative values represent performance below average.

The innovation on pesticide application technique is important and urgent for orchard management due to the rapid decrease in agricultural labor and the increase in labor costs. Intelligent pesticide application techniques should be developed to cope with the current dilemma in orchard management (He, 2018). However, different pesticide application techniques have their own characteristics (Figure 6), and there is still no satisfactory application technique to date. UAV application technology could significantly improve the working efficiency, which has been recognized by most researchers (Li et al., 2021b). It also had a significant advantage in pollution control and water resource reduction during pesticide application due to its low-volume spraying. Higher working efficiency also led to lower carbon dioxide emissions from the machine itself. However, spraying uniformity was the greatest challenge for UAV application technology, which could directly affect the effectiveness of pest control in orchard management (Qin et al., 2018). A previous study had also showed that spraying performance of UAV technique was much poorer than conventional methods, especially in spray uniformity and penetration (Wang et al., 2022). Even in field crops application with lower canopy, the spray performance and pest control effectiveness under UAV application were still unsatisfied (Li et al., 2021b). The research emphasis of UAV application technology should focus on improving the application effect and reducing drifting pollution. Nozzle improvement, droplet control, airflow control and operation parameter optimization are of great significance in improving the application effectiveness of UAV technology (Wang et al., 2022). For the orchard manager, UAV spraying technology could only be considered if the spraying uniformity and penetration have been improved and can achieve better performance in pest or disease control.

Fewer studies had evaluated the performance of UGV application technique in orchard management in past years. Interestingly, our study proved that UGV application technology also has advantages in working efficiency improvement, water savings and carbon dioxide emission reduction compared to conventional methods. UGV application technology had a better spraying uniformity with the price of higher pesticide pollution, which differed from UAV spraying technology. For orchard managers, UGV application technology could be a better choice for the replacement of conventional methods considering the stable application performance and low natural resource waste. However, how to control environmental pollution during actual practice is also important to the development of UGV application technology in the future. It is possible to reduce environmental pollution and increase pesticide utilization efficiency through the adjustment of flow and spray angle. On the other hand, considering the cost of intelligent equipment and the planting scale of orchards, conventional spray gun technology could also be a valid choice. However, with orchard standardization and scale management, intelligent pesticide application techniques will play an important role in the future of orchard management. Different scales and types of orchards could influence the performance of different pesticide application techniques. To improve the application performance and environmental pollution of intelligent application techniques, we should also pay more effort in better detection sensors together with more accurate machine learning algorithms. Precision application technology which is based on monitoring sensors and recognition algorithms, as well as control of spray parameters will play an important role in intelligent equipment in the future.

This study systematically clarified the characteristics of intelligent unmanned vehicle techniques in pesticide application from the aspects of working effectiveness, efficiency, environmental pollution, water saving and carbon dioxide reduction, which are important for the development of intelligent equipment in orchard. It should be noted that our study was conducted in a standardized orchard. To cover a wider range of working environments, we will further consider the effects of orchard type, planting scale, ecological area, and other factors on the performance of different pesticide application techniques. Moreover, a meta-analysis could also help to achieve more accurate and universal results. Meanwhile, all intelligent equipment in this study were operated manually which may cause various among receptions. As the development of automatic navigation and application in UAV and UGV, the artificial error can be avoided for further research.

## Conclusion

This study clarified the characteristics of different pesticide application techniques from the aspects of working effectiveness, efficiency, environmental pollution and resource protection. UAV application techniques have advantages of high working efficiency and low environmental pollution and natural resource consumption. However, it performed worse in spray performance compared to the UGV and conventional manual methods. UGV application techniques could improve spray performance at the cost of high environmental pollution. The conventional spray gun technique also showed good spray performance. However, the tradition method was unfriendly to environmental protection and the green development of agriculture. Intelligent pesticide application techniques could be an alternative to conventional methods. Improving spray performance and controlling environmental pollution are major directions for UAV and UGV technology improvement in the future.

Further research should be undertaken to investigate the comprehensive performance of intelligent application techniques in different kinds of orchards. A meta-analysis can be carried out to make the results more accurate and universal. This study comprehensively evaluated the characteristics of different pesticide application techniques and put forward suggestions for the development of orchard plant protection in the future, providing scientific support for the transition of intelligent management in orchards and the development of smart agriculture in China.

## Data availability statement

The raw data supporting the conclusions of this article will be made available by the authors, without undue reservation.

## Author contributions

YJ performed most of the experiments with the assistant of TL, PQ, CY, and FC provides guidance of work while XH and JS contributed to the study conception and design. All authors contributed to the article and approved the submitted version.

## Funding

This work was supported by the National Natural Science Foundation of China (no. 31761133019), China Agriculture Research System of MOF and MARA (CARS-28), and the 2115 Talent Development Program of China Agricultural University.

## Conflict of interest

The authors declare that the research was conducted in the absence of any commercial or financial relationships that could be construed as a potential conflict of interest.

## Publisher’s note

All claims expressed in this article are solely those of the authors and do not necessarily represent those of their affiliated organizations, or those of the publisher, the editors and the reviewers. Any product that may be evaluated in this article, or claim that may be made by its manufacturer, is not guaranteed or endorsed by the publisher.
